# Effects of physical training programs on female tennis players’ performance: a systematic review and meta-analysis

**DOI:** 10.3389/fphys.2023.1234114

**Published:** 2023-08-17

**Authors:** Nuannuan Deng, Kim Geok Soh, Borhannudin Abdullah, Dandan Huang, He Sun, Wensheng Xiao

**Affiliations:** ^1^ Department of Sports Studies, Faculty of Educational Studies, Universiti Putra Malaysia, Selangor, Malaysia; ^2^ College of Physical Education, Chongqing University, Chongqing, China; ^3^ School of Physical Education Institute (Main Campus), Zhengzhou University, Zhengzhou, China; ^4^ Department of Sports Sciences, Huzhou University, Huzhou, China

**Keywords:** training program, athletic performance, intervention, exercise, tennis players

## Abstract

**Background:** Tennis is among the world’s most popular and well-studied sports. Physical training has commonly been used as an intervention among athletes. However, a comprehensive review of the literature on the effects of physical training programs on female tennis players’ performance is lacking. Therefore, this systematic review and meta-analysis aimed to determine the effects of physical training on performance outcomes in female tennis players.

**Methods:** A comprehensive search was conducted on Web of Science, PubMed, SPORTDicus, Scopus, and CNKI from inception until July 2023 to select relevant articles from the accessible literature. Only controlled trials were included if they examined the effects of physical training on at least one measure of tennis-specific performance in female tennis players. The Cochrane RoB tool was employed to assess the risk of bias. The CERT scale was used to examine the quality of program information. The GRADE approach was adopted to evaluate the overall quality of the evidence. The Comprehensive Meta-Analysis software was used for the meta-analysis.

**Results:** Nine studies were selected for the systematic review and seven for the meta-analysis, totaling 222 individuals. The study’s exercise programs lasted 6–36 weeks, with training sessions ranging from 30 to 80 min, conducted one to five times per week. Muscle power (ES = 0.72; *p* = 0.003), muscle strength (ES = 0.65; *p* = 0.002), agility (ES = 0.69; *p* = 0.002), serve velocity (ES = 0.72; *p* = 0.013), and serve accuracy (ES = 1.14; *p* = 0.002) demonstrated significant improvement following physical training, while no notable changes in linear sprint speed (ES = 0.63; *p* = 0.07) were detected.

**Conclusion:** Although research on physical training in sports is diversified, studies on training interventions among female tennis players are scarce. This review found that existing training programs yield some favorable outcomes for female tennis players. However, further research with high methodological quality is warranted on the tailoring of specific training programs for female tennis players. There should be more consistent measuring and reporting of data to facilitate meaningful data pooling for future meta-analyses.

## Introduction

Technical proficiency, tactical awareness, psychological skill, and physical condition are closely connected to tournament success in tennis (Kovacs, 2007; Reid et al., 2007). Previous studies provide essential data on the match-play demands of tennis players (Kovacs, 2006; Reid and Schneiker, 2008; Fernandez-Fernandez et al., 2014; Pluim et al., 2023). Tennis is an intermittent sport in which participants need to perform high-intensity movements such as decelerations, accelerations, direction changes, and strokes over a variable duration (Kovacs, 2007). During rallies, points are scored in less than three to 10 seconds on average (Dobos et al., 2021), with sprints of eight to 15 m and three to four directional changes (Genevois, 2019). Tennis players are widely regarded as needing greater levels of physical performance in order to execute complex shots and compete well against more elite opponents (Ulbricht et al., 2016). Specifically, competitive tennis players need a combination of physical traits such as speed and agility, as well as excellent aerobic fitness, to attain high-level performance (Kovacs, 2007; Fernandez-Fernandez et al., 2009; Fernandez-Fernandez et al., 2012). These elements enable a player to perform an instantaneous explosion, a rapid turn and stop, make posture adjustments, and confront intense situations (Kovacs, 2007). Moreover, it seems that reaching optimal physical performance is a fundamental necessity for achieving strong stroke performance, such as high ball speed, which is required for success in a game of tennis (Baiget et al., 2019). Furthermore, a variety of factors such as technique, flexibility, muscular strength, and power affect the final functional performance of an elaborate chain of torque transfers (e.g., a groundstroke or serve in tennis) (Fisher and Steele, 2014; Fett et al., 2020; Turner et al., 2022). Therefore, the main emphasis in training competitive tennis players should be enhancing players’ capacity to execute high-intensity actions and quickly recover them consistently (Reid et al., 2016).

In the literature, most experimental research tends to focus on the performance of male tennis players (Fernandez-Fernandez et al., 2012; Kilit and Arslan, 2019; Yildiz et al., 2019). Moreover, Xiao et al. (2021) conducted a systematic review of exercise training for young tennis players’ physical fitness. However, all the reviewed studies only examined male participants. Women remain to be under-represented in the evidence base, with menstrual cycle complications noted as a key obstacle to include female athletes in such trials (McNulty et al., 2020). As a result, information gathered from male athletes is often applied to the female population (Emmonds et al., 2019; Elliott-Sale et al., 2021). Nevertheless, given the biological differences (e.g., menstrual cycle, hormonal profile), applying studies undertaken on male athletes to female athletes may be inappropriate (Hughes et al., 2023). In recent years, there has been an exponential increase in the participation, professionalism, and visibility of female sports (International Olympics Committee, 2023). Coaches, sports scientists, and researchers aim to discover effective techniques to improve performance and provide advantages in elite sports (Strahorn et al., 2017). An ideal approach would offer positive adaptations produced by training without inducing excessive physical strain (Strahorn et al., 2017). However, hormonal variations throughout the menstrual cycle create several confounding factors that affect female tennis players’ performance, making research design and subsequent interpretation of data challenging (Meignié et al., 2021). Meanwhile, there is a lack of agreement on whether various physical training approaches effectively improve female tennis players’ performance. Consequently, a more comprehensive literature analysis on physical training in female tennis players is essential. Conducting a systematic review and meta-analysis is imperative and timely to statistically combine the findings from various investigations and examine the impact of physical training programs on performance outcomes in female tennis players. In addition to enhancing the study’s statistical power, a meta-analysis can provide more precise data and address the inconsistencies observed in previous experimental investigations (Liu et al., 2022). This study aimed to achieve three primary objectives:

1. Collect data on physical training programs used by female tennis players.
2. Integrate the current information on these programs and present a summary of the characteristics of the selected studies.
3. Recognize deficiencies within the current literature and propose potential areas for future research and practical implications.

## Methodology

The research team of this study followed the guidelines outlined in the updated Preferred Reporting Items for Systematic Reviews and Meta-Analysis (PRISMA) statement (Page et al., 2021). This protocol was registered (registration number: CRD42023428947) at the international prospective register of systematic reviews (PROSPERO).

### Eligibility criteria

This review included academic literature published in either English or Chinese without limitations on the publication year. The studies needed to meet the criteria stated in the PICOS framework to be considered for analysis (McKenzie et al., 2019).

Table 1 depicts the inclusion and exclusion criteria for the systematic review and meta-analysis. Articles that satisfied all the conditions below were considered for inclusion: 1) participants were female tennis players, without any restrictions on age, tennis experience, or competition level; 2) the control group was in a regular tennis program; 3) the evaluation of physical performance areas (e.g., strength, power, agility) or key performance indicators (e.g., ball speed) was documented; and 4) the physical training program was conducted without restrictions on the specific type of exercise employed. This encompassed a wide range of physical training methods, including but not limited to the following:

-Resistance training refers to physical conditioning approaches that incorporate the gradual application of a broad range of resistive loads, variable movement velocities, and a number of training modalities (Faigenbaum et al., 2013).
-Plyometric training is characterized by fast and powerful movements that require muscles to undergo lengthening, immediately followed by rapid shortening in what is known as the stretch-shortening cycle (Clark et al., 2016).
-Core training refers to an exercise regimen that uses one’s own body weight to balance the spine and strengthen the core muscles (McGill, 2010).
-Neuromuscular training could be defined as training enhancing unconscious motor responses by stimulating both afferent signals and central mechanisms responsible for dynamic joint control (Risberg et al., 2001).
-Functional training is defined as a progressive series of exercises that instruct participants on how to manage their body weight in all planes of movement (Santana, 2015).
-Coordination training refers to a program designed to improve an individual’s ability to synchronize and control their body movements effectively (Tsetseli et al., 2010). Usually, coordination exercises are suggested to be practiced during the early phases of growth and development (i.e., childhood and adolescence) (Radwan, 2014; Padrón-Cabo et al., 2020).

**TABLE 1:** Eligibility criteria according to the PICOS conditions.

Category	Inclusion criteria	Exclusion criteria
Population	Female tennis players	Female tennis players with health problems (e.g., injuries, recent surgery)
Intervention	The minimum duration for physical interventions was set at 2 weeks	Interventions not involving physical training or exercise interventions involving physical training program in conjunction with other psychological training interventions
Comparison	Active control group	Absence of active control group
Outcome	At least one tennis performance outcome, association with physical performance areas or key performance indicators	Lack of baseline and/or follow-up data
Study design	RCT or non-RCT	Cross-sectional studies, case studies, articles not written in English or Chinese

RCT, randomized control trial; non-RCT, non-Randomized control trial.

Studies were excluded if 1) no original data was reported (protocol, review, patent, and letter; 2) they were conducted without separating males and females; 3) the participants were unhealthy or injured; 4) there was no control group; 5) no outcome of any tennis performance was included; and 6) the exercise intervention was combined with other psychological interventions (e.g., mental imagery training; cognitive training).

### Search strategy and selection process

On 23 March 2023 and updated on 22 July 2023, a systematic search was carried out to retrieve articles related to the topic from five electronic databases: Web of Science, SPORTDiscus, PubMed, SCOPUS, and CNKI. A Boolean search syntax using the operators “AND” and “OR” was applied. The keywords “training”, “exercise*”, “intervention”, “physical training”, “exercise training”, “resistance training”, “strength training”, “aerobic exercise*“, “power training”, “fitness training”, endurance training, “conditioning training”, “physical therapy”, “female”, “woman”, “women”, “girl”, “tennis” were utilized. An example of a PubMed search is as follows {[“training” (Title/Abstract) OR “exercise*” (Title/Abstract) OR “intervention” (Title/Abstract) OR “physical training” (Title/Abstract) OR “exercise training” (Title/Abstract) OR “resistance training” (Title/Abstract) OR “strength training” (Title/Abstract) OR “aerobic exercise*” (Title/Abstract) OR “power training” (Title/Abstract) OR “fitness training” (Title/Abstract) OR “endurance training” (Title/Abstract) OR “conditioning training” (Title/Abstract) OR “physical therapy” (Title/Abstract)] AND [“female” (Title/Abstract) OR “woman” (Title/Abstract) OR “women” (Title/Abstract) OR “girl” (Title/Abstract)]} AND [“tennis” (Title/Abstract)]. Furthermore, a manual search was performed on Google Scholar and references to ensure no pertinent articles were overlooked. Meanwhile, the data-gathering process was supported by skilled librarians to ensure its accuracy and completeness.

**FIGURE 1:**
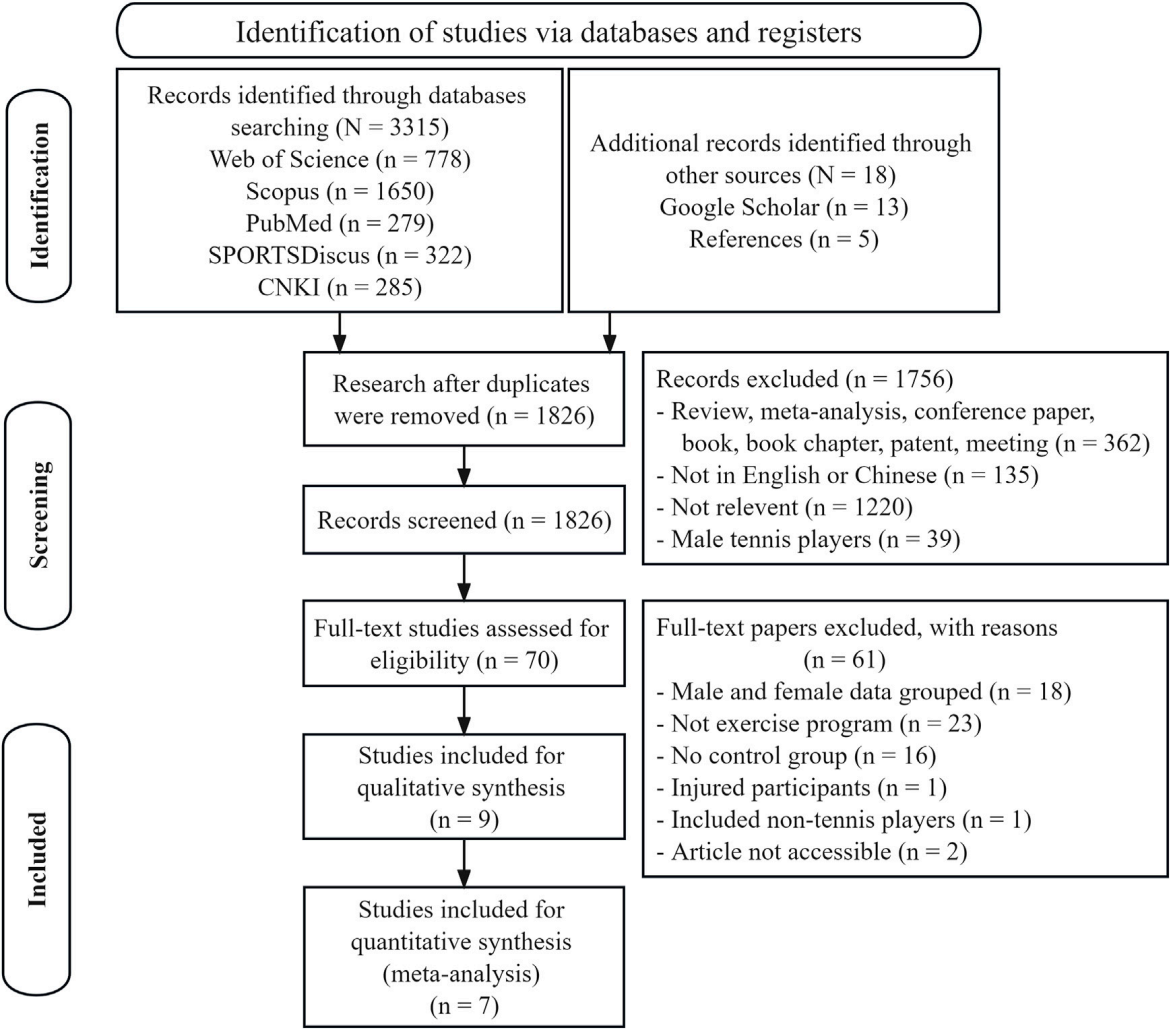
Flow diagram of the study selection.

The specific steps involved in the study’s selection are shown in Figure 1. Initially, duplicate articles were removed. The second stage involved assessing the title and abstract. The full-text screening procedure included evaluating predetermined eligibility criteria. Two independent reviewers (ND and DH) completed this procedure. Any dispute was explored further. Where necessary, a third reviewer (KGS) participated until consensus was obtained.

### Data extraction

The information from each study was obtained by two reviewers (ND and DH) using a Microsoft Excel spreadsheet (Microsoft Corporation, Redmond, WA, United States), and a third reviewer (KGS) verified its accuracy. The data considered were:

1. Publication year, name of the first author;
2. Players’ characteristics: sample size, age, competition level, and tennis experience;
3. Characteristics of the training protocol (e.g., frequency, intensity);
4. Measures index and study outcomes.

### Risk of bias and the certainty of evidence assessment

By scrutinizing the study design of each included article, we found that all of them were randomized controlled trials. Therefore, we employed the Revised Cochrane Risk of Bias tool for randomized trials (RoB 2.0) to assess the risk of bias in each paper. The tool categorizes risk bias into five categories, and each was assigned a rating from “low risk of bias”, “some concerns of bias”, or “high risk of bias,” based on the specific signaling questions in RoB 2.0. Ultimately, an overall assessment of biased judgment was made for each study. The reviewers (ND and HD) adhered to the guidelines set forth by the Cochrane community. Additionally, the other two reviewers (KGS and BA) utilized the Consensus on Exercise Reporting Template (CERT), which comprises a 19-item checklist with seven categories, to assess the suitability of exercise intervention descriptions and reporting (Slade et al., 2016). A score of <9 is considered“low”methodological quality, and a score of ≥9 is considered“high”.

The Grading of Recommendations, Assessment, Development, and Evaluations (GRADE) was used together with the online tool “GRADEpro” to verify the certainty of the evidence, accounting for the studies’ limitations, such as the risk of bias, inconsistency of training program effects, indirectness, imprecision, or other factors (Schünemann et al., 2020). Two reviewers (ND and DH) assessed the certainty of the evidence and the risk of bias. The review team, which consisted of experts in systematic review methodology (KGS and BA), verified the results. In case of disagreements, further discussion was held among the team to resolve them.

### Data synthesis and analysis

According to the Cochrane Handbook (Higgins et al., 2022), meta-analyses can be conducted with as few as two studies (Valentine et al., 2010). However, low sample sizes are common in the field of physical training (Nygaard Falch et al., 2019; Su et al., 2022; Deng et al., 2023). Therefore, we performed meta-analyses when ≥3 studies (or experimental groups) with available baseline and follow-up data for the same measure were available. These studies were consolidated for meta-analysis using Comprehensive Meta-Analysis software (version three; Biostat, Englewood, NJ, United States). Conversely, a narrative synthesis was conducted to compile and analyze the findings (Deng et al., 2022). A random-effects analysis model for continuous data was utilized through an inverse variance statistical method during the meta-analyses to determine the effect sizes (ES; Hedge’sg) and 95% confidence intervals (CIs) of performance outcome measures between groups. The specified data entry format comprised “means, standard deviation (SD) pre and post, sample size (n) in each group, pre/post correlation”. The data was standardized by post-score SD values since no research revealed correlation and it could not be computed with high precision (Borenstein et al., 2021). Standardizing the data in this way obviates the requirement for correlation values when calculating ESs. In cases where studies presented data in formats other than means and SD (e.g., range, median, or standard error values), we followed the suggested approach as outlined in prior research (Higgins and Deeks, 2011; Wan et al., 2014; Lee and Lee, 2015) and performed appropriate conversions. The data for the different formats was inputted using Comprehensive Meta-Analysis software. If the necessary information was unavailable in the original article or supplementary materials, we attempted to contact the authors. In instances when we received no response to our inquiries, we employed the Graph Digitizer software (Digitizelt, Germany) to extract relevant data from figures or graphs. However, if the required data was inaccessible through these means, the study was excluded from the meta-analysis. The ES values and their corresponding 95% CIs were reported. The magnitudes of the ES were evaluated using the following range: <0.2, trivial; 0.2–0.6, small; >0.6–1.2, moderate; >1.2–2.0, large; >2.0–4.0, very large; >4.0, extremely large (Hopkins et al., 2009). The control group was proportionally divided when examining studies involving multiple experimental groups to enable effective participant comparisons (Higgins et al., 2008). The I^2^ statistic was utilized to evaluate heterogeneity and varying degrees of heterogeneity were classified as low, moderate, and high. Specifically, heterogeneity was considered low when it was 25% or below, moderate from 25% to 75%, and high when it exceeded 75% (Higgins et al., 2003). The extended Egger’s test (Egger et al., 1997) was utilized to evaluate the studies’ publication bias risk. Furthermore, a sensitivity analysis was conducted when Egger’s test yielded significant results. The threshold for statistical significance was established at *p* < 0.05.

## Results

### Study selection

As shown in Figure 1, 3,315 papers were discovered through databases, while an additional 18 studies were found through Google Scholar and references. After manually removing duplicates, 1826 records remained. These records’ titles and abstracts were reviewed. Ultimately, 70 papers were eligible for full-text analysis. After conducting a full-text examination, 61 publications were excluded, and nine papers that met all inclusion criteria for the systematic review were retained. Seven of the nine papers were incorporated into the meta-analysis.

### Risk of bias and the certainty of evidence assessment

**FIGURE 2:**
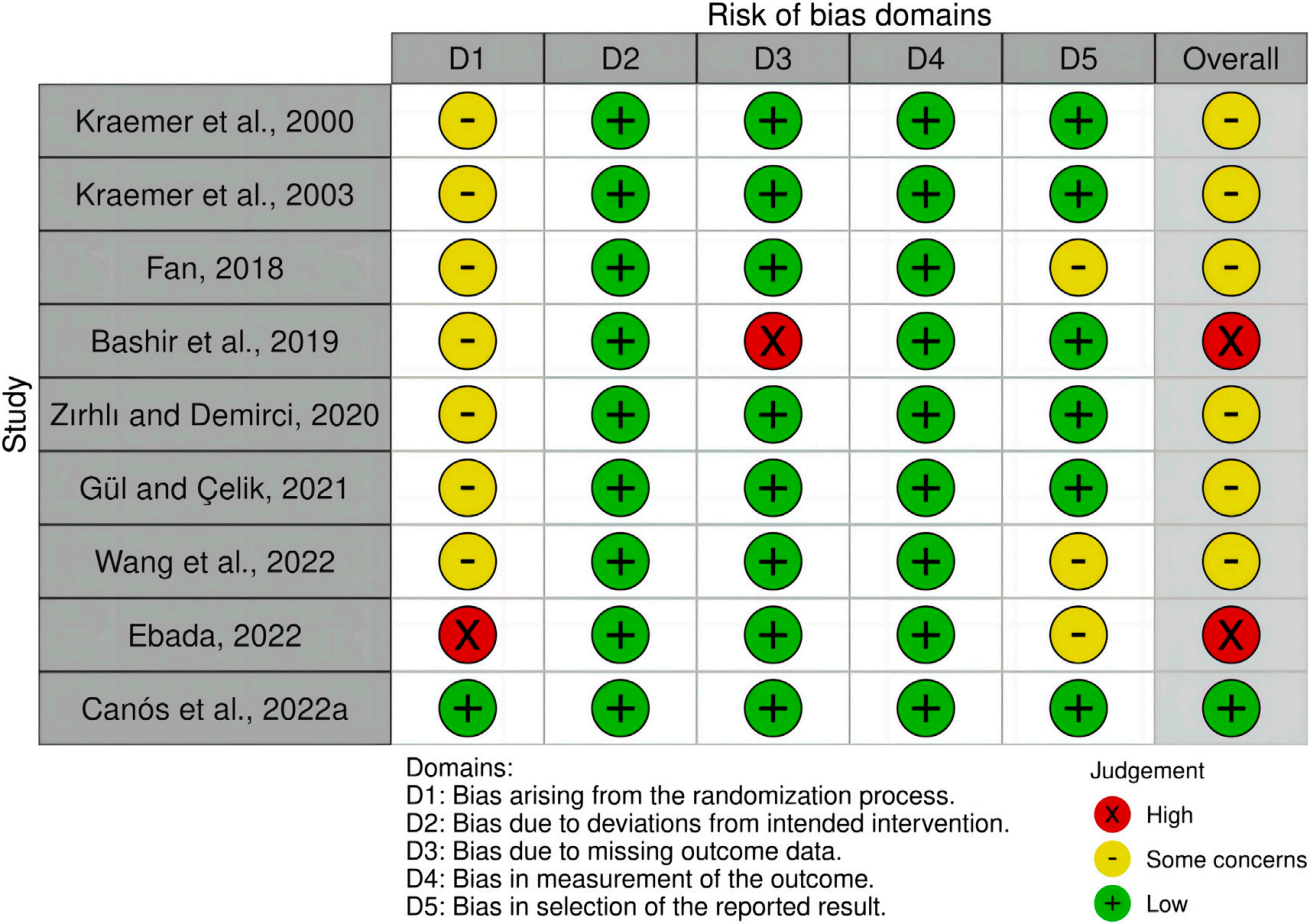
Risk of bias summary for each included study. *Created using Robvis (visualization tool).

**FIGURE 3:**
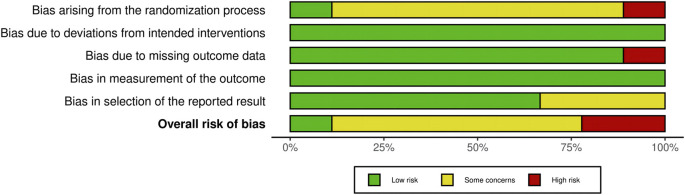
Risk of bias. *Created using Robvis (visualization tool).

The findings of the RoB-2 evaluations are presented in Figures 2, 3. Only one study outlined a technique for creating randomization sequences using stratified block randomization (Canós et al., 2022b). One study (Ebada, 2022) had a high-risk bias arising from randomization due to insufficient reporting of the randomization process and baseline data. Another trial found a high-risk bias associated with missing data caused by a dropout rate of approximately 10% (Bashir et al., 2019). Six studies had preregistered protocols, but three had concerns about bias in selecting the reported results (Fan, 2018; Ebada, 2022; Wang et al., 2022). Additionally, concerning the reporting and description of the exercise interventions, the included trials fulfilled an average of 6.6 out of 19 items on the CERT scale. Among them, three studies were graded as “high,” (Kraemer et al., 2000; Kraemer et al., 2003; Canós et al., 2022a), and six studies were graded as “low” (Fan, 2018; Bashir et al., 2019; Zırhlı and Demirci, 2020; Gül and Çelik, 2021; Ebada, 2022; Wang et al., 2022) (Table 2).

**TABLE 2:** Results of the methodological quality evaluation of the scale Consensus on Exercise Reporting Template (CERT).

CERT items	Kraemer et al. (2000)	Kraemer et al. (2003)	Fan (2018)	Bashir et al. (2019)	Zırhlı and Demirci (2020)	Gül and Çelik (2021)	Canós et al. (2022b)	Wang et al. (2022)	Ebada (2022)
1. Description of the type of exercise equipment	Yes	Yes	Yes	Yes	Yes	Yes	Yes	Yes	Yes
2. Description of the qualifications, expertise and/or training	No	No	Yes	No	No	No	No	No	No
3. Describe: exercises are individual or in a group	Yes	Yes	Yes	Yes	Yes	Yes	Yes	Yes	Yes
4. Describe: exercises are supervised or unsupervised; how they are delivered	Yes	Yes	No	No	No	No	Yes	No	No
5. Detailed description of how adherence to exercise is measured and reported	Yes	Yes	Yes	Yes	No	No	Yes	No	No
6. Detailed description of motivation strategies	No	No	No	No	No	No	No	No	No
7a. Detailed description of the decision rule(s) for determining exercise progression	Yes	Yes	Yes	No	No	No	No	No	No
7b. Detailed description of how the exercise program was progressed	Yes	Yes	Yes	No	Yes	No	Yes	No	Yes
8. Detailed description of each exercise to enable replication	Yes	Yes	No	No	Yes	No	Yes	No	Yes
9. Detailed description of any home program component	No	No	No	No	No	No	No	No	No
10. Describe whether there are any non-exercise components	No	No	No	No	No	No	No	No	No
11. Describe the type and number of adverse events that occur during exercise	No	No	No	No	No	No	Yes	No	No
12. Describe the setting in which the exercises are performed	Yes	No	Yes	No	No	No	No	Yes	No
13. Detailed description of the exercise intervention	Yes	Yes	No	Yes	Yes	Yes	Yes	Yes	Yes
14a. Describe whether the exercises are generic (one size fits all) or tailored	Yes	Yes	No	No	No	No	No	No	No
14b. Detailed description of how exercises are tailored to the individual	Yes	Yes	No	No	No	No	No	No	No
15. Describe the decision rule for determining the starting level	Yes	Yes	No	No	No	No	No	No	No
16 a. Describe how adherence or fidelity is assessed/measured	No	Yes	No	No	No	No	Yes	No	No
16 b. Describe the extent to which the intervention was delivered as planned	No	No	No	No	No	No	No	No	No
Total score (0–19)	10	12	7	4	5	3	9	4	5

The summary of the findings table (Supplementary Appendix S1) reveals the evaluation of the level of certainty of the evidence regarding the subsequent performance for different outcomes. Initially, all studies were rated as high certainty as randomized controlled trials. However, after evaluating each domain, the overall certainty was downgraded due to the significant risk of bias, imprecision, or inconsistency. Based on the GRADE evaluation, the evidence’s certainty level is considered low to very low.

### Population characteristics

The pertinent details concerning the characteristics of the studies are presented in Table 3. The total participant count amounted to 222 individuals. The age of the players ranged from 9.25 ± 0.74 (Gül and Çelik, 2021) to 22.6 ± 3.2 years (Fan, 2018). Concerning players’ expertise level, four studies employed regional or local sport club players (Bashir et al., 2019; Zırhlı and Demirci, 2020; Gül and Çelik, 2021; Canós et al., 2022b), while one study examined national players (Wang et al., 2022). Three studies recruited collegiate players (Kraemer et al., 2000; Kraemer et al., 2003; Fan, 2018), and one study selected beginners (Ebada, 2022). Additionally, seven studies reported between 1 year and 9 years of specific-tennis experience. However, two papers did not mention players’ experience (Ebada, 2022; Wang et al., 2022).

**TABLE 3:** Characteristics of included studies.

References	Population characteristics	Training protocols		Measures index	Outcomes
		EG	CG		
Kraemer et al. (2000)	N = 24; age: EG1, 19.0 ± 0.9 years; EG2, 18.9 ± 1.2 years; CG:19.8 ± 1.7 years; Level: collegiate; TE: 7.8 ± 2.4 years	- Type: periodized resistance training (EG1)	- Type: Tennis training and conditioning training	Body composition (body mass, body fat, fat-free mass); Strength (Bench press, shoulder press, leg press); SV	EG1: body mass↑, body fat↑, fat-free mass↑, bench press↑, shoulder press↑, leg press↑, SV↑, EG2: body mass→, body fat→,fat-free mass→, bench press↑, shoulder press↑, Leg press↑, SV→
		- Frequency/time/length: 2–3 days/90 min s/9 months			
		- Volume: 2-4sets × 4–15 reps			
		- Rest: 1–2 min for 8–15 reps, 2–3 min for 4–6 reps			
		- Intensity: maximal			
		- Progression: volume	- Frequency/time/length: 2–3 days/90 min/9 months		
		- Type: single-set resistance training (EG2)			
		- Volume: 1 set × 8–10 reps			
		- Rest: 1–2 min between sets			
		- Intensity: maximal			
Kraemer et al. (2003)	N = 27; age: EG1, 19.2 ± 1.1 year; EG2 18.6 ± 1.3 years; CG 19.3 ± 1.6 years; Level: collegiate; TE: 8.1 ± 3.5 years	- Type: non-linear periodized resistance training (EG1)	- Type: tennis training and conditioning training	Body composition (body mass, body fat, fat-free mass); Strength (hand grip, leg press, bench press, shoulder press); Endurance (VO_2max_); Speed (10m, 20 m); Agility (LAT); Power (VJH); SV	EG1: body mass→, body fat↑, fat-free mass↑, 10 m →, 20 m→, LAT →, VJH →, grip strength; leg press↑, bench press↑, shoulder press↑, VO2max↓, SV↑, FV↑, BV↑, EG2: body mass→, body fat↑, fat-free mass↑, 10 m →, 20 m→, LAT →, VJH →, handgrip; leg press↑, bench press↑, shoulder press↑, VO_2max_↓, SV↑
		- Frequency/time/length: 3 days/90 min s/9 months			
		- Volume: 1-3sets, 4–15 RM training loads			
		- Rest: 1.5–3 min s between sets			
		- Intensity:low to high*			
		- Progression: volume, intensity			
		- Type: non-periodized resistance training (EG2)	- Frequency/time/length: 3 days/90 min/9 months		
		- Frequency/time/length: 3 days/90 min/9 months			
		- Volume: 1-3sets, 8–10 RM training loads			
		- Rest: 1.5–2 min between sets			
		- Intensity: moderate*			
Fan (2018)	N = 24; age: EG1, 22.3 ± 2.2; EG2, 22.6 ± 3.2 years; CG, 21.9 ± 2.9 years; Level: collegiate; TE: > 8 years	- Type: core training (EG1); core training with resistance training (EG2)	- Type: tennis drills	SA	EG1 = EG2: SA↑
		- Frequency/time/length: 3days/90 min s/6 weeks	- Frequency/time/length: 5 days/120 min s/6 weeks		
		- Progression: volume, type of drill, intensity			
Bashir et al. (2019)	N = 30; age: 15.3 ± 0.8 years; Level: regional; TE: > 1 year	- Type: core training + regular training	- Type: regular tennis training	Dynamic balance (SEBT); agility (*t*-test)	SEBT ↑, *t*-test↑
		- Frequency/time/length: 3 days/NR/5 weeks	- Frequency/time/length: 3 days/NR/5 weeks		
		- Volume: 1–4 sets × 6–20reps			
Zırhlı and Demirci (2020)	N = 20; age: 11.20 ± 0.834 years; level: tennis clubs; TE: > 2 years	- Type: functional training + regular training	- Type: tennis stroke drills	Speed (10-m); power (VJH); flexibility (SAR); strength (hand grip); agility (*t*-test)	10-m↑,VJH↑, SAR↑, hand grip↑, *t*-test↑
		- Frequency/time/length: 2days/90 min/8 weeks	- Frequency/time/length: 4 days/90 min s/8 weeks		
		- Volume: 3 sets × 7–10reps	- Intensity: estimated power intensity of 75%		
		- Rest: 3 min s between sets			
		- Intensity: 75% maximum heart rate			
Gül and Çelik 2021	N = 16; age: 9.25 ± 0.74 years; level: tennis club; TE: 9.25 ± 0.74 years	- Type: coordination training + club tennis training	- Type: club tennis training	Power (VJ); agility (*t*-test); speed (20 m)	VJ ↑, *t*-test ↑, 20 m→
		- Frequency/time/length: 5 days/60 min s/8 weeks	- Frequency/time/length: 5 days/60 min/8 weeks		
Wang et al. (2022)	N = 40; age: EG, 18.2 ± 1.9 years; CG, 17.9 ± 2.2 years; level: national second- level; TE: NR	- Type: core training	- Type: traditional strength training	Strength (bridge-type test, abdominal fatigue test); SA; SV	Bridge test↑, abdominal fatigue test↑, SA→, SV↑
		- Frequency/time/length: 3 days/30 min/9 weeks	- Frequency/time/length: 3 days/30 min/9 weeks		
Ebada (2022)	N = 17; age: EG, 12.17 ± 0.4 years; CG, 12.09 ± 0.6 years; level: beginners, TE: NR	- Type: plyometric training	- Type: normal tennis training	Strength (hand grip); power (MBT)	Hand grip↑, MBT↑
		- Frequency/time/length: 3 days/50 min s/7 weeks	- Frequency/time/length: 3 days/50 min/7 weeks		
		- Volume: 3-5sets × 5–20 reps			
		- Rest: 30 s between sets			
		- Progression: volume			
Canós et al. (2022a)	N = 24, age: EG1, 15.6 ± 1.0 years; EG2, 15.8 ± 0.7 years; CG, 15.6 ± 0.9 years; level: tennis club; TE: >7 years	- Type: machine-based neuromuscular training + tennis and injury prevention drills (EG1)	- Type: tennis and injury prevention drills	Power (CMJ, OMBT, FMBT, BMBT); speed (5m, 10m, 15 m); agility (5-0-5 test); SV	EG1: CMJ↑, OMBT→, FMBT→, BMBT →, 5 m↑, 10 m↑, 15 m→, 5-0-5 test→; SV↑, EG2: CMJ↑, OMBT↑, FMBT↑, BMBT↑, 5 m↑, 10 m↑, 15 m→, 5-0-5 test ↑; SV→
		- Frequency/time/length: 3 days/120 min/8 weeks			
		- Volume: 3 sets × 6–8 reps			
		- Rest: 1.5 min s between sets, 3 min between round			
		- Intensity: 50%–70% 1RM	- Frequency/time/length: 3 days/120 min/8 weeks		
		- Type: flywheel-based neuromuscular training + tennis and injury prevention drills (EG2)			
		- Frequency/time/length: 3 days/120 min/8 weeks			
		- Volume: 3 sets × 6–8 reps			
		- Rest: 1.5 min between sets, 3 min between round			

TE, tennis experience; Freq, frequency; Reps, repetitions; maximal, involving either maximal effort to achieve maximal height, distance, reactive strength index, velocity (time contact or fast stretch-shortening cycle), or another marker of intensity.

For the studies marked with an *, the intensity was reported only qualitatively; 1 RM, repetition maximum; VJH, vertical jump height; SEBT, star excursion balance Test; SAR, sit and reach test; SLJ, standing Long Jump; OMBT, overhead medicine-ball throws; FMBT, forehand medicine-ball throws; BMBT, backhand medicine-ball throws; LAT, lateral agility test; SV: serve velocity; SA, serve accuracy; →, no significant changes; ↑, significant changes.

### Interventions characteristics

A total of 13 intervention programs were employed in the included studies. These programs encompassed resistance training (Kraemer et al., 2000; Kraemer et al., 2003), core training (Fan, 2018; Bashir et al., 2019; Wang et al., 2022), a combination of core exercise with resistance training (Fan, 2018), functional training (Zırhlı and Demirci, 2020), coordination training (Gül and Çelik, 2021), neuromuscular training (Canós et al., 2022a), and plyometric training (Ebada, 2022). The trials’ duration in nine studies varied from five to 9 weeks, with two studies conducting long-term investigations lasting 9 months. Furthermore, most works incorporated three to five training sessions per week, with a duration ranging from 30 to 120 min, 1 to 5 sets per exercise, 5 to 20 repetitions per set, and 30–180s of rest between sets. Four studies reported the training intensity, using different indices (e.g., heart rate; one-repetition maximum). More detailed descriptions of intervention characteristics can be found in Table 3.

### Qualitative synthesis

Table 3 summarizes the findings for each of the included studies.

### Physical performance outcome

Two studies examined the effects on body composition indices, including fat-free mass, body fat, and body mass. In both studies, the participants and intervention characteristics were similar, and the training techniques comprised different forms of resistance training, including periodized and single-set resistance training (Kraemer et al., 2000) and non-linear periodized and non-periodized resistance training (Kraemer et al., 2003). At four, six, and 9 months, these workouts substantially reduce body fat percentage and fat-free mass (*p* < 0.05). Nevertheless, no significant changes associated with body mass were identified (*p* > 0.05). Moreover, no differences in body composition characteristics were detected between experimental groups (EGs) at any time (Kraemer et al., 2000; Kraemer et al., 2003).

Five studies investigated performance power. In Kraemer et al. (2003) study, female tennis players underwent different neuromuscular training modes, and it was concluded that there was no significant difference in vertical jump height between the EGs. Conversely, Zırhlı and Demirci (2020) discovered a significant change in the vertical jump height of tennis players who had received functional training, as there were considerable differences between their pre-test and post-test values. Gül and Çelik (2021) discovered that 8-week coordination training sessions significantly increased standing long jump performance. Ebada (2022) demonstrated that plyometric training significantly improved power performance compared to regular tennis drills, as measured by the medicine ball throw test. Additionally, Canós et al. (2022b) showed that both EG1 and EG2 methods substantially improved lower body power as measured by countermovement jump tests. Still, there were no significant changes in medicine ball throw tests.

Four studies examined female tennis players’ muscle strength. Kraemer et al. (2000) utilized two different 9-month resistance training programs. Significant improvements (*p* < 0.01) were observed in the shoulder press, leg press, and bench press tests for both experimental groups. According to Kraemer et al. (2003), non-linear and non-periodized resistance training significantly impacted the hand grip, leg press, bench press, and shoulder press (*p* < 0.05). Zırhlı and Demirci (2020) evaluated EG and CG utilizing an 8-week functional training program. They discovered that hand grip strength increased considerably (*p* < 0.05). Similarly, Ebada (2022) investigated hand grip strength among female tennis players; their results revealed that a 7-week plyometric training program greatly enhanced hand grip test scores. Wang et al. (2022) demonstrate that 9 weeks of core ability training positively impacted female tennis players’ core strength (e.g., abdominal fatigue test).

Four studies analyzed linear sprint speed. Kraemer et al. (2003) discovered that sprinting speed remained unchanged in EG1 and EG2, with no notable differences between groups in 10 m or 20 m sprinting speeds after four, six, and 9 months of training. One study implemented functional training in young female tennis players and found that the 8-week functional training program, along with routine tennis training, positively impacted the 10 m sprint speed of female tennis players (*p* < 0.05) (Zırhlı and Demirci, 2020). Gül and Çelik (2021) found that coordination drills applied to tennis players for 8 weeks increased the speed parameter (20 m) (*p* = 0.045). Canós et al. (2022a) researched the effect of different modes of neuromuscular training on the sprint times (5, 10, and 15 m) of young female tennis players at baseline at week four and week eight. At week four, there was a moderate to very large improvement in sprint time at 5 m and 10 m in EG1 and EG2. Significant decreases in the 10-m sprint time were observed in EG1 (*p* = 0.011) and EG2 from weeks four to eight. However, no significant group-by-time interaction was observed at 15 m (*p* = 0.274).

Five studies were conducted on agility. Kraemer et al. (2003) discovered that 9 months of resistance training significantly impacted agility (lateral agility test) (*p* < 0.01). Similarly, Bashir et al. (2019) discovered that 5 weeks of core training markedly enhanced agility (*t*-test). Gül and Çelik (2021) found that agility improved significantly after 8 weeks of coordination drill sessions (*t*-test). Zırhlı and Demirci (2020) compared EG and CG using an 8-week functional training program, and they found that agility performance (*t*-test) significantly improved in the EG. Additionally, Canós et al. (2022b) compared the effects of two different neuromuscular training protocols (EG1 vs EG2) on agility performance (5-0-5 test) over 8 weeks, and both protocols resulted in significantly improved agility (*p* < 0.05).

Endurance was evaluated in one study (Kraemer et al., 2003), which examined endurance using the VO_2max_ test. Surprisingly, VO_2max_ decreased significantly after 9 months of resistance training programs. One study was related to balance. A significant difference in dynamic balance was found after the 5-week core training program (Bashir et al., 2019). Another study evaluated flexibility using the sit and reach test. Based on the results, 8 weeks of functional training significantly impacted flexibility.

### Tennis-specific skills outcome

Four studies examined the ball velocity of female tennis players, with the majority focusing on measuring the serve velocity. Kraemer et al. (2000) discovered that the periodized training group experienced a significant increase in serve velocity at post-tests. In contrast, no significant changes were found in the single-set or control groups. Kraemer et al. (2003) demonstrated that serve velocity increased considerably during EG1 and EG2 sessions. However, the percentage gains in this tennis stroke were much greater following EG1 after 9 months. Wang et al. (2022) found that 9 weeks of core training were superior to conventional strength training for improving the serve velocity of tennis players. Canós et al. (2022a) demonstrated that machine-based neuromuscular training significantly increased serve velocity from baseline to week eight (*p* = 0.040). Nevertheless, the results indicated no changes in the EG2.

Two studies that evaluated female tennis players’ serve accuracy had contradictory results. In one study, two 6-week core training programs were conducted, and the results showed that players who participated in core exercises plus resistance workouts or who solely did core training significantly improved their serve accuracy (*p* < 0.05). In contrast, Wang et al., ‘s 2022 study, which involved a 9-week core training program for female tennis players, showed no significant enhancement in serve accuracy (*p* > 0.05).

### Meta-analyses

The meta-analysis was performed on specific trials assessing muscle power, strength, linear sprint speed, agility, serve velocity and accuracy. However, several other outcomes required further pooling data and were excluded from the meta-analysis. The data utilized for the meta-analyses can be found in Supplementary Appendix S2.

**FIGURE 4:**
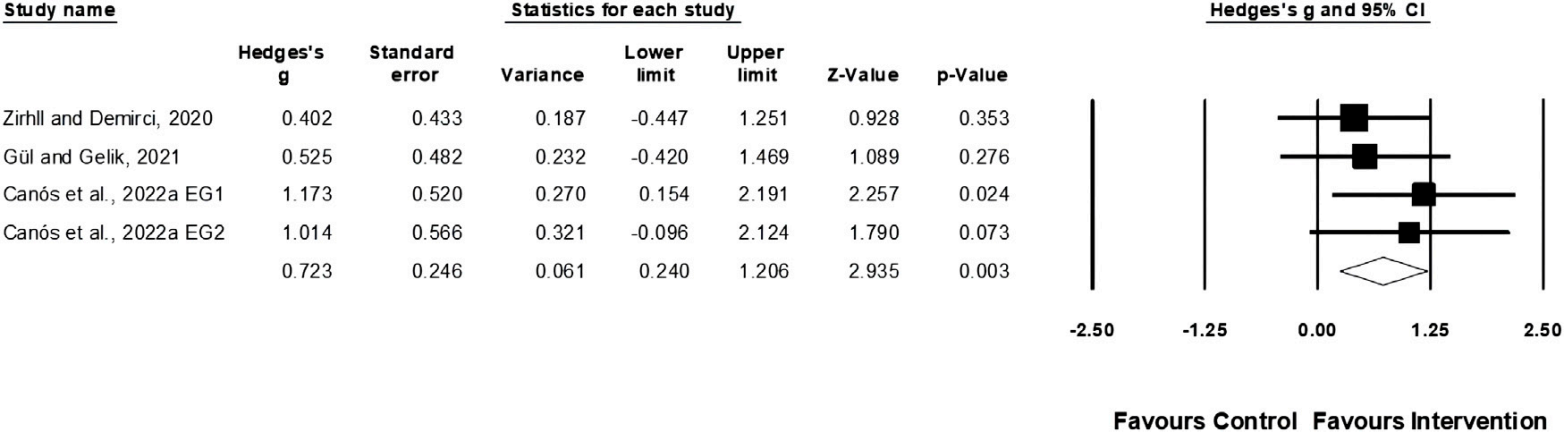
Forest plot of changes in muscle power performance in female tennis players participating in training intervention compared to controls. Values shown are effect sizes (Hedges’s g) with 95% confidence intervals (CI). The size of the plotted squares reflects the statistical weight of the study. EG = experimental group.

Four studies provided data for muscle power, involving five experimental and three control groups (pooled n = 87). Egger’s test indicated a *p* = 0.002. Following a sensitivity analysis, the exclusion of one study (Kraemer et al., 2003) resulted in an Egger’s test *p* ≥ 0.05. Ultimately, three studies of three studies of four experimental and three control groups were included for analysis. Physical training moderately affected muscular power performance (ES = 0.72; 95% CI = 0.24–1.21; *p* = 0.003; *I* ^*2*^ = 0.0%; Figure 4).

**FIGURE 5:**
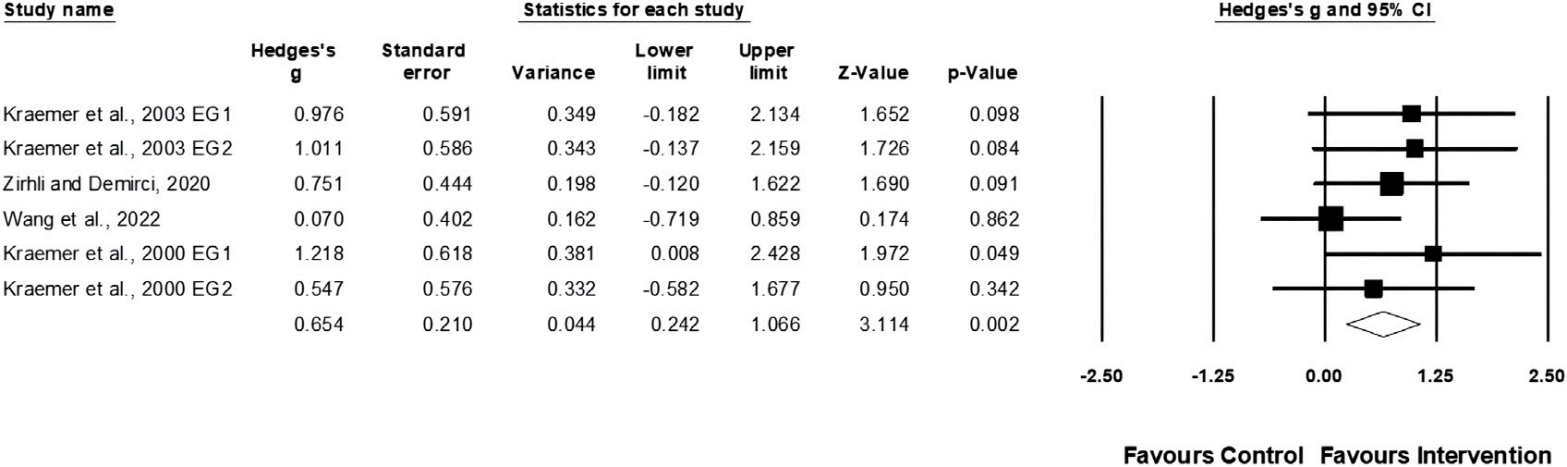
Forest plot of changes in muscle strength performance in female tennis players participating in training intervention compared to controls. Values shown are effect sizes (Hedges’s g) with 95% confidence intervals (CI). The size of the plotted squares reflects the statistical weight of the study. EG = experimental group.

Four studies provided data for muscle strength, involving six experimental and four control groups (pooled n = 93). PT had a moderate effect on muscle strength performance (ES = 0.65; 95% CI = 0.24–1.07; *p* = 0.002; *I* ^*2*^ = 0.0%; Egger’s test *p* = 0.050; Figure 5).

**FIGURE 6:**
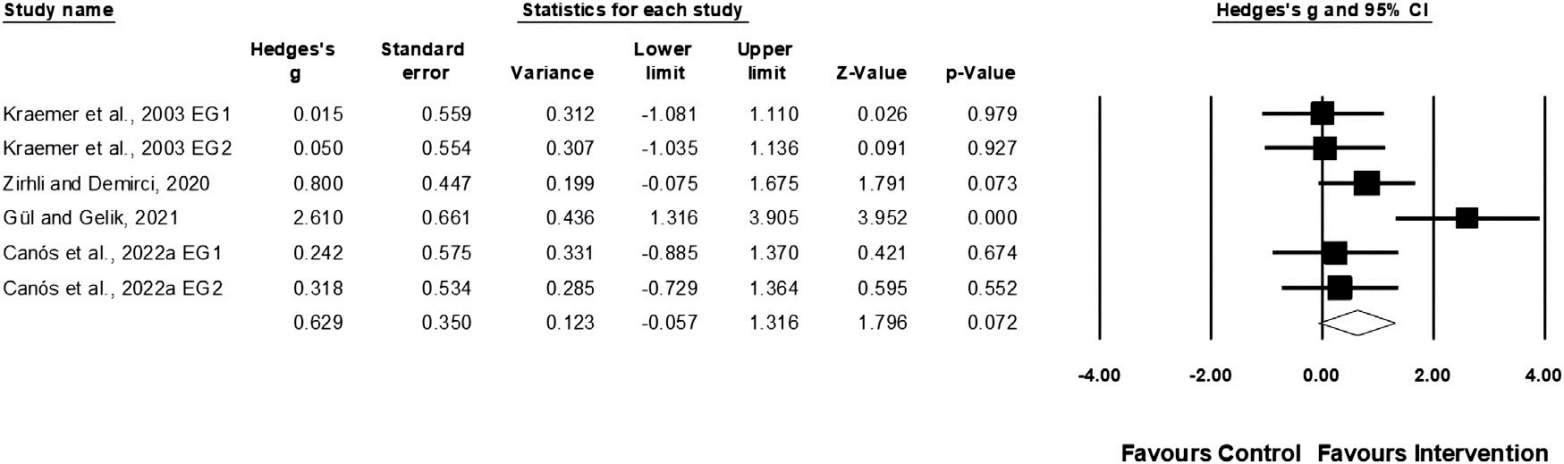
Forest plot of changes in linear sprint speed performance in female tennis players participating in training intervention compared to controls. Values shown are effect sizes (Hedges’s g) with 95% confidence intervals (CI). The size of the plotted squares reflects the statistical weight of the study. EG = experimental group.

Four studies involving six experimental and four control groups provided data for linear sprint speed (pooled n = 87). Physical training moderately affected linear sprint speed performance (ES = 0.63; 95% CI = −0.06–1.32; *p* = 0.07; *I* ^*2*^ = 59.04%; Egger’s test *p* = 0.474; Figure 6). Despite a positive effect size, the meta-analysis revealed no statistically significant improvement in linear sprint speed following the interventions.

**FIGURE 7:**
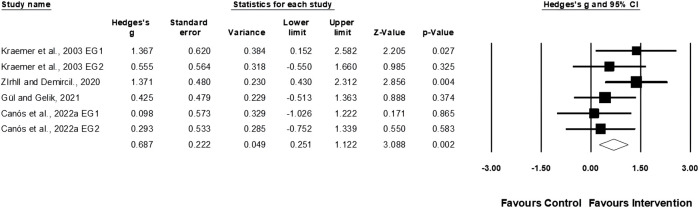
Forest plot of changes in agility performance in female tennis players participating in training intervention compared to controls. Values shown are effect sizes (Hedges’s g) with 95% confidence intervals (CI). The size of the plotted squares reflects the statistical weight of the study. EG = experimental group.

Four studies involving six experimental and four control groups provided data for agility (pooled n = 87). Physical training moderately affected agility performance (ES = 0.69; 95% CI = 0.25–1.12; *p* = 0.002; *I* ^*2*^ = 3.58%; Egger’s test *p* = 0.922; Figure 7).

**FIGURE 8:**
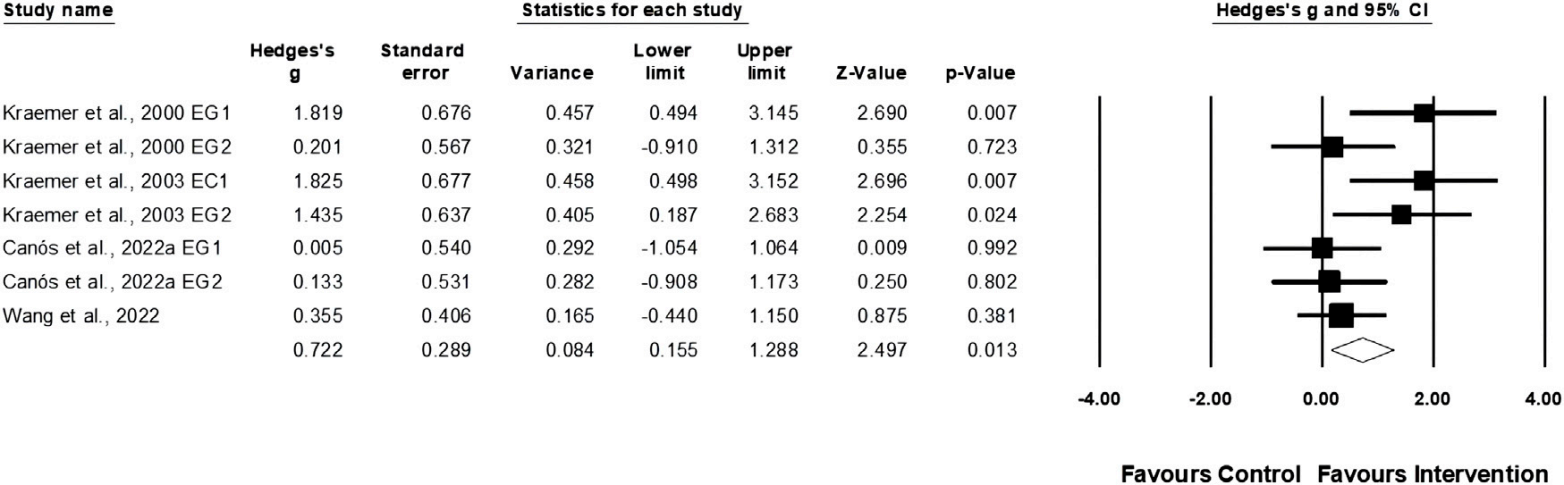
Forest plot of changes in serve velocity performance in female tennis players participating in training intervention compared to controls. Values shown are effect sizes (Hedges’s g) with 95% confidence intervals (CI). The size of the plotted squares reflects the statistical weight of the study. EG = experimental group.

Four studies involving seven experimental and four control groups provided data for serve velocity (pooled n = 115). Physical training moderately affected the tennis serve velocity performance (ES = 0.72; 95% CI = 0.16–1.29; *p* = 0.013; *I* ^*2*^ = 45.7%; Egger’s test *p* = 0.07; Figure 8).

**FIGURE 9:**
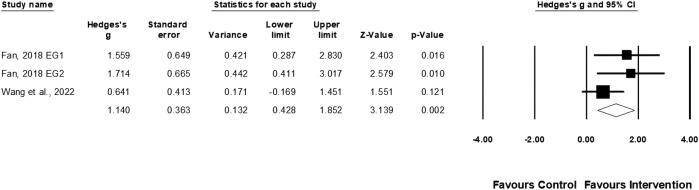
Forest plot of changes in serve accuracy performance in female tennis players participating in training intervention compared to controls. Values shown are effect sizes (Hedges’s g) with 95% confidence intervals (CI). The size of the plotted squares reflects the statistical weight of the study. EG = experimental group.

Two studies involving three experimental and two control groups provided data for serve accuracy (pooled n = 59). Physical training greatly affected serve accuracy performance (ES = 1.14; 95% CI = 0.43–1.85; *p* = 0.002; *I* ^*2*^ = 22.6%; Egger’s test *p* = 0.058; Figure 9).

## Discussion

This review analyzed nine papers to determine the effects of physical training (e.g., resistance training, core training) on female tennis players’ physical performance and tennis-specific skills. The results indicated that these exercises significantly positively impacted several measured variables. Nonetheless, given the restricted number of studies incorporated in the analysis, the strength of the conclusions derived from the meta-analysis could be more robust. Furthermore, the evidence reported in this review was classified as very low to low levels for the measured variables, suggesting that caution should be exercised when interpreting the outcomes.

### Effect on physical performance

The body composition of athletes is essential to their physical condition and overall health (Santos et al., 2014). Body composition analysis in athletes may aid in optimizing competitive performance and monitoring the effectiveness of training regimes (Ackland et al., 2012). Resistance training is considered the most effective strategy for building lean muscle mass (Wewege et al., 2022). One of the included studies found that employing a periodized multiple-set training strategy for resistance training yielded superior outcomes compared to a single-set exercise regimen in terms of body composition measures (i.e., body mass, body fat, and fat-free mass) in competitive female tennis players (Kraemer et al., 2000). According to Draper and Marshall’s (2014) study, there were significant associations between the body mass of female tennis players and their rankings. However, another study included in this review found that the resistance training groups showed no significant increase in body mass (Kraemer et al., 2003). Of note, the number of sets in a resistance training program has been a pivotal point of debate and discussion in the literature (Galvão and Taaffe, 2004). Bottaro et al. (2011) found that neither a training stimulus of one nor three sets was sufficient for developing muscle thickness. Nevertheless, Radaelli et al. (2015) noted that single and multiple-set training groups increased fat-free mass and reduced body fat percentage with no significant differences. Previous research suggests a potential relationship between exercise intensity and training volume in terms of their impact on changes in body composition. For example, Irving et al. (2008) suggested that high-intensity endurance training might be an effective stimulus for inducing favorable changes in body composition (e.g., body weight, fat mass). In the case of resistance exercise, low-volume, high-intensity training routines appear to be the most time-efficient method for increasing muscle mass (Carpinelli et al., 2004). Slentz et al. (2004) also discovered a dose-response association between training volume and body fat loss, indicating that a greater amount of exercise leads to larger reductions. In addition, several studies have indicated that effective improvement in body composition can be achieved through the use of plyometric training, high-intensity interval training, functional training, or a combination of these approaches (Racil et al., 2016; da Silva et al., 2017; Alonso-Fernández et al., 2017; Sultana et al., 2019; Ramirez-Campillo et al., 2022). However, the optimal training parameters to enhance body composition are less evident. Further studies are warranted to investigate the interaction between training parameters (e.g., type, volume, intensity) on changes in body composition.

Tennis strokes are widely recognized to be impacted by muscle power (Palmer et al., 2018; Fett et al., 2020). The power of a tennis player is directly linked to the speed of their racket head and the ball’s velocity (Kovacs et al., 2008). The current meta-analysis shows that physical training interventions can enhance muscle power in female tennis players. Neuromuscular training (Canós et al., 2022b), functional training (Zırhlı and Demirci, 2020), and coordination training (Gül and Çelik, 2021) are among the exercise types that can improve power in female tennis players. Neuromuscular training might be especially beneficial for female athletes, as they typically have lower baseline levels of lower extremity power than male athletes (Myer et al., 2005). One included study (Canós et al., 2022a) compared machine-based and flywheel-based neuromuscular training in power tests using medicine ball throws. The findings indicated that flywheel-based neuromuscular training exhibited greater effectiveness than machine-based training. This superiority could be attributed to the emphasized eccentric phase of contraction and the utilization of energy storage inherent in flywheel-based exercises (Canós et al., 2022b). Moreover, Adami et al. (2022) provided support for the claim that athletes who engage in functional training develop greater muscular power. Wakeling et al. (2010) emphasized the importance of muscle coordination in achieving efficient limb movements and optimal power output. From this point of view, coordination exercises can assist players in synchronizing and utilizing their muscles effectively, reducing unnecessary movements and energy loss (Joyce and Lewindon, 2014) and increasing power production. Although Kraemer et al. (2003) did not find significant improvement in muscle power, resistance training remains one of the most effective methods for enhancing power output in athletes (Reid and Schneiker, 2008; Harries et al., 2012; Morris et al., 2022). However, as mentioned earlier, the outcomes of resistance training programs can be influenced by various factors such as intensity, volume, and exercise selection. In addition, previous research has recommended the ballistic six exercises (i.e., plyometric training) program for overhead-throwing athletes, including commonly used power exercises (Pretz, 2004; Carter et al., 2007; Girard and Millet, 2009). Ebada (2022) reported significant improvement in muscular power after a ballistic six exercises program. Thus, ballistic six exercises may be an appropriate intervention for improving the neuromuscular ability to enhance upper body power (Turgut et al., 2019) in female tennis players. However, further research is needed to confirm this notion.

Muscle strength is vital during rallies for executing tennis-specific footwork and powerful strokes (Dobos et al., 2021). Muscles and joints require strength to elevate performance (e.g., ball velocity) and prevent injury (e.g., tendons and ligaments, protection of joints) (Kovacs, 2006). The results of the present meta-analysis indicate that exercise interventions can enhance muscle strength among female tennis players. The two studies showed a significant effect of physical training on strength, utilizing different resistance drills (Kraemer et al., 2000; Kraemer et al., 2003; Lyakh et al., 2011). For athletes involved in resistance training, developing muscle strength is a common goal as it determines the force muscles can generate under specific conditions and plays a vital role in athletic performance (Williams et al., 2017). Multiple studies in the literature have shown that performing multiple sets of resistance exercises is more effective in enhancing strength than practicing a single set (Kelly et al., 2007; Fröhlich et al., 2010; Bottaro et al., 2011). Although single-set resistance programs can result in notable strength gains, they are not as effective as multiple-set programs. Single-set programs may be recommended when training time is limited (Galvão and Taaffe, 2004). According to Wolfe et al. (2004), adult athletes provide evidence that using single-set conventional resistance training may be suitable in the early stage of resistance training. However, if athletes aim to enhance their muscle strength further, they should incorporate multiple-set conventional resistance training programs (Lesinski et al., 2016). Similarly, Kraemer et al. (2003) findings align with a previous meta-analysis indicating that periodized resistance training programs are more effective than non-periodized programs at promoting strength gains (Williams et al., 2017). Other studies showed that functional training (Zırhlı and Demirci, 2020), core training (Wang et al., 2022), and plyometric training (Ebada, 2022) could help tennis players improve their strength. Zırhlı and Demirci (2020) suggested that functional training may be as effective as conventional resistance training in increasing muscular strength. Also, Xiao et al. (2021) recent review endorses functional training methods for athletes seeking to improve their muscular strength. Furthermore, several studies and meta-analyses support the notion that plyometric exercise effectively enhances athletes’ muscular strength (de Villarreal et al., 2010; Oxfeldt et al., 2019; Ramirez-Campillo et al., 2020; Ramirez-Campillo et al., 2021). In addition, core muscles are crucial for stabilizing the spine and trunk during exercise and improving leg balance and athletic performance (Bliss and Teeple, 2005). Previous investigations have confirmed that core training can improve athletes’ trunk strength (Hibbs et al., 2008; Ozmen and Aydogmus, 2016).

When playing tennis, the time between winning and losing strokes is minuscule. Therefore, even a second or millisecond delay during a sprint can hinder a player’s performance (Önal and Aras, 2022). A player with better sprint performance can reach the ball faster, affording them more time to prepare for a shot (Kramer et al., 2021). However, our meta-analysis revealed no statistically significant differences in linear sprinting speed between intervention and control groups. In tennis gameplay, after serving the ball with high velocity, a tennis player needs to accelerate not only in a straight line but also laterally and in multiple directions (Moya-Ramon et al., 2020). Previous research has identified linear sprinting speed as a key indicator of the ability to change direction (Sheppard and Young, 2006; Shaikh and Mondal, 2012). The selected studies in the current review employed multiple types of training methods to improve female tennis players’ sprint speed (5–20 m), including functional training (Zırhlı and Demirci, 2020), resistance training (Kraemer et al., 2003), coordination training (Gül and Çelik, 2021), and neuromuscular training (Canós et al., 2022b). Sprint speed was assessed using different devices, including photo-electric cells (Kraemer et al., 2003; Gül and Çelik, 2021), a stopwatch (Zırhlı and Demirci, 2020), and timing gates (Canós et al., 2022a). However, the majority of study findings revealed that the training strategy had no statistically significant impact on female tennis players’ linear sprint speed. de Villarreal et al. (2012) proposed that the lack of specificity of the exercises to sprinting may have been responsible for the absence or small improvements in sprint times. The authors highlighted that incorporating training programs with a focus on greater horizontal acceleration, such as sprint-specific plyometric exercises and jumps with horizontal displacement, would lead to optimal gains in sprint speed performance. Moreover, it is widely understood that optimizing the benefits of physical training is mostly dependent on the manipulation of program factors such as volume, duration, rest interval, and intensity (Kraemer and Ratamess, 2004; Boullosa et al., 2020; Iversen et al., 2021). Furthermore, monitored sprint times over very short distances can exhibit variations of up to 50%–60% due to differences in methodology and equipment (Haugen and Buchheit, 2016). The use of manual timing and photocells may lead to significant absolute errors during short sprint distances (≤20 m) (Haugen and Buchheit, 2016). In addition, it has been said that improvements in sprinting performance may not always show up immediately after a period of strength training (Moir et al., 2007). Consequently, athletes might require a certain amount of time to acclimate and effectively apply the gains in strength to the actual act of sprinting (Moir et al., 2007). It is also possible that the results of the included studies may have been influenced by the menstrual cycle (Hughes et al., 2023). However, it is worth noting that the limited number of studies included (n = 4) does not provide enough evidence to establish definite conclusions regarding the actual impact of physical training on sprint performance among female tennis players. Therefore, further research is necessary to delve deeper into the role of physical training methods in influencing linear sprint speed in female tennis players.

In tennis, agility pertains to a player’s capacity to promptly and reactively alter their direction in response to the tennis ball’s movement or path (Young and Farrow, 2006). Our meta-analysis found a noteworthy difference in agility between the control and intervention groups. Specifically, the data demonstrated that various training methods, such as functional training (Zırhlı and Demirci, 2020), core training (Bashir et al., 2019), coordination training (Gül and Çelik, 2021), and flywheel-based neuromuscular training (Canós et al., 2022b) could effectively enhance agility among female tennis players. However, a study utilizing two types of resistance training (Kraemer et al., 2003) found no significantly improved agility for both EGs. Although a previous systematic review accompanied by meta-analysis research concluded that resistance training is an efficient method to enhance agility, the findings suggested that resistance training might have a more significant impact on agility among males and youth compared to females and adults (Chaabene et al., 2020). As mentioned earlier, the absence of improvement in agility among female tennis players after a training program may be influenced by various aspects, including training-related factors (e.g., intensity), or subject-related factors (e.g., menstrual cycle). Agility can be developed through training that emphasizes changes in direction, underlying physical qualities, as well as perceptual and decision-making factors (Zouhal et al., 2019). Besides, developing the lower limbs’ reactive force can also improve agility (Brughelli et al., 2008). In the literature, Yildiz et al. (2019) found that the functional model was more successful than the conventional training model in improving agility performance. Moreover, a recent review provides evidence that the neuromuscular training program is valuable for improving athletes’ agility (Akbar et al., 2022). Furthermore, core stabilization is crucial in the early stages of motor development. The central nervous system activates the muscles in the trunk before movement, creating a stable foundation to anticipate the forces generated by the limbs (Hoppe et al., 2014). For players to effectively perform the change of direction movements, they must have core stability to withstand sheer forces on the spine, especially during multidirectional tasks (Okada et al., 2011). Additionally, agility is strongly associated with motor coordination and relies on the neurophysiological organization of movement and the coordination of different degrees of freedom (Marković et al., 2007). Exercises that promote coordination have the potential to enhance agility (González-Fernández et al., 2021). However, few EGs have evaluated the same training strategy in this context, making it difficult to identify the most effective form of physical training for improving agility performance.

Adequate endurance capacity (e.g., VO_2max_) is essential for tennis players during rallies and rest periods between rallies. This capacity facilitates more efficient recovery (Karnia et al., 2010). Despite this, only one study in the current review investigated tennis players’ endurance levels (Kraemer et al., 2003). The results found that resistance training led to a notable decrease in VO_2max_. This decline in aerobic capacity may be attributed to a shift in the body’s focus toward adaptations for increased force generation and tolerance of glycolytic exercise metabolism (Kraemer et al., 1995). Moreover, one study evaluated dynamic balance using the star excursion balance test (Bashir et al., 2019). Maintaining an appropriate level of balance is fundamental for success in tennis. It not only enhances training effectiveness (Malliou et al., 2010; Caballero et al., 2021) but also reduces the risk of lower limb injuries such as ligament sprains and muscle strains (Pluim et al., 2006). In the current review, Bashir et al. (2019) study indicated a statistically significant effect of core training on balance in female tennis players. Improved core stability may enhance performance by providing a robust base for generating greater force in the upper and lower body (Tarnanen et al., 2012), thereby positively impacting dynamic balance. By improving dynamic balance, athletes can cultivate a more resilient and secure foundation that facilitates improved lower body movements (Barrio et al., 2022). Similarly, only one study explored flexibility (Zırhlı and Demirci, 2020). Flexibility is required in tennis as players sometimes hit the ball in extreme body positions (Kovacs et al., 2016). Zırhlı and Demirci (2020) concluded that implementing a functional training regimen substantially and favorably impacts female tennis players’ flexibility levels. The Optimum Performance Pyramid emphasizes that good functional movement is essential for achieving a complete range of motion and efficient power utilization (Cook, 2003). Due to limited literature on the subject, it is challenging to draw firm conclusions about the efficacy of current training approaches under investigation by researchers. Therefore, additional research is necessary to acquire a more comprehensive understanding of the impact of physical training on these variables.

### Effect on tennis-specific skills

In tennis, the technique is commonly associated with two critical factors: ball speed and accuracy (Kolman et al., 2019). Even if a player’s shot is accurate, a lack of speed could potentially disadvantage them against their opponent (Landlinger et al., 2012). The meta-analysis results showed that exercise interventions significantly and moderately affect serve velocity in female tennis players. Body mass has shown strong relations to serve velocity in female participants (Fernandez-Fernandez et al., 2019). Kraemer et al. (2000) research support the notion that body mass is critical to serve velocity. Resistance training programs that target the enhancement of shoulder joint strength, which is essential during tennis strokes, have yielded favorable results in boosting serve velocity (Colomar et al., 2022). Moreover, Fan (2018) indicated that core exercise combined with resistance exercise training could be more effective than core training alone for enhancing tennis serve velocity in female tennis players. Luo et al. (2022) have already demonstrated the positive benefits of core training on athletic skill performance. A well-developed core facilitates force transmission from the lower to the upper body, increasing energy efficiency throughout the body (Stephenson and Swank, 2004). However, according to some researchers, achieving better performance requires a combination of various training methods rather than relying solely on core training (Smart et al., 2011; Dinç and Ergin, 2019). Canós et al. (2022a) compared two types of neuromuscular training programs (machine-based vs. flywheel-based). The results revealed that only the machine-based training program significantly enhanced serve velocity. In contrast, a previous study that used similar neuromuscular training programs showed no changes in serve velocity for male tennis players (Canós et al., 2022b). Therefore, it remains uncertain whether machine-based training is more effective than flywheel training.

Skilled players can hit the ball with accuracy and power, allowing them to precisely aim it at their opponent’s side of the court (Elliott, 2006). Previous research showed a correlation between ball accuracy and tennis performance level (Landlinger et al., 2012). The present meta-analysis showed that core training interventions can significantly promote serve accuracy compared with controls. Training programs often prioritize augmenting muscle strength across the entire kinetic chain while maintaining serve accuracy to enhance serve performance in tennis (Roetert et al., 2009). The mechanisms of core training can account for these findings. A solid and stable core is crucial for effective movement control and action output (Kibler et al., 2006). Core strength training focuses on enhancing the connection between nerves and muscles, developing smaller muscle groups, and improving coordination among active, auxiliary, and antagonist muscles (Borghuis et al., 2008). The greater the core area’s strength, the higher the body’s stability and precision of technical movements during tennis matches (Press, 2009). Consequently, core training can aid in improving or sustaining serve accuracy. While Fernandez-Fernandez et al. (2016) observed that plyometric training resulted in significant improvements in serve accuracy among male tennis players, the limited research leaves uncertainty regarding whether other training programs, such as plyometrics, have the same effect on female tennis players.

### Practical applications

Due to the limited available literature, reliable recommendations could not be drawn. Nevertheless, this review can establish a direction for future research and practice. Existing training methods have been shown to enhance the physical performance of female tennis players in terms of muscle strength, muscle power, agility, serve velocity and serve accuracy. The training programs in the studies ranged from 30 to 120 min, with 3–5 weekly training sessions lasting between 3 and 36 weeks. However, more specialized training may be required if the objective is to improve female tennis players’ linear sprint speed. Moreover, many critical aspects of tennis-specific performance, such as reaction time, coordination, and groundstroke, still need to be explored. Furthermore, the effects of other intervention components, such as high-intensity interval training and plyometric jump training (Salonikidis and Zafeiridis, 2008; Girard et al., 2018; Fernandez-Fernandez et al., 2019), have been extensively discussed by researchers and recommended for improving tennis performance, but exclusively for male tennis players. Therefore, one recommendation for future research exploring the impact of physical exercise on female tennis players’ performance is to enhance the quality of study designs and implement more specific, well-designed training practices.

### Limitation and future direction

The studies encompassed in this review offer valuable insights into the experiences of female athletes, yet they do have some limitations. Firstly, the review included a relatively limited number of available studies (n = 9). Due to the scarcity of studies, conducting subgroup analyses based on physical training type, frequency, length, time, and participants’ age was not feasible, as there were fewer than three studies available for at least one of these moderators. Moreover, the analysis of the effects of physical training on outcomes such as balance, flexibility, and endurance was precluded. Secondly, the review only covered two languages, which may have hindered the comprehensiveness of the results. Thirdly, some studies lacked a thorough description of the training protocol. For instance, in four out of nine papers, the intensity of the training program was not specified. Future researchers are recommended to adopt a more rigorous methodological approach and ensure comprehensive reporting, including details on factors such as volume and intensity, when implementing training interventions. Notably, the most significant limitation of this meta-analysis, which pertains specifically to female athletes, lies in the methodological considerations. The significance of the menstrual cycle’s impact on physical performance is increasingly acknowledged as a crucial aspect of women’s sports and an area that requires further investigation (Carmichael et al., 2021). Nevertheless, none of the studies included in this analysis mentioned incorporating the menstrual cycle of female tennis players into their procedures, making it challenging to provide evidence-based recommendations. It remains unclear whether these studies implemented the necessary methodological considerations, such as addressing the known physiological differences between the sexes, particularly concerning menstrual cycle phases (Elliott-Sale et al., 2021). Prior studies have shown that natural hormonal fluctuations during the menstrual cycle can impact physiological responses and training adaptations (Sung et al., 2014; Meignié et al., 2021). Therefore, it is essential to take into account menstrual cycle phases as a parameter when developing individualized training strategies. Finally, the maturation of athletes might play a significant role in the overall training effect, as adaptations are believed to be more substantial during and after peak height velocity, which is the period when adolescents experience their fastest growth in stature (Romero et al., 2021). Recently, Fernandez-Fernandez et al. (2023) conducted a study showing that the maturity stage influenced physical performance in a large sample of young tennis players. They found that post-peak height velocity players outperformed both pre- and circa-peak height velocity peers in linear sprints, jumping ability, and agility tests. However, among the included studies of this review, five of them enlisted young female tennis players without describing their biological maturity. To address this concern in future research involving youth female tennis athletes, it is crucial to measure maturity status through methods such as skeletal age, Tanner stages, or anthropometric assessments, and systematically report these findings. By doing so, future studies can provide more accurate insights into the adaptability of young athletes to exercise.

## Conclusions

The findings of this review indicate that current physical training interventions had a moderate to large impact on physical and tennis-specific skills in female tennis players, including muscle power, muscle strength, agility, serve velocity, and serve accuracy. However, no significant effect on linear sprint speed was observed. It is crucial to exercise caution when interpreting these results due to the evidence’s low to very low certainty level. Meanwhile, there was insufficient evidence for several key performances (e.g., balance, flexibility, and endurance) of female tennis players. While studies on physical training are more diverse in sports, those conducted on training interventions among female tennis players are scarce. Further research with high methodological quality is warranted on the tailoring of specific training programs for female tennis players. There should be more consistent measuring and reporting of data to facilitate meaningful data pooling for future meta-analyses.

## Data Availability

The original contributions presented in the study are included in the article/Supplementary Material, further inquiries can be directed to the corresponding author.
