# Supplementary material for: Effects of physical training programs on female tennis players’ performance: a systematic review and meta-analysis
Source: Front Physiol. 2023 Aug 17;14:1234114. doi: 10.3389/fphys.2023.1234114 (PMC10470022; doi:10.3389/fphys.2023.1234114)
Supplement: Supplementary file 1 [file Table1.DOCX]

**Table S1.** Summary of findings of RCTs comparing the effects of physical training on performance outcome of female tennis players after 5 to 9 weeks.

| Outcomes | Certainty assessment | | | | | No of participants and studies | Certainty of evidence  (GRADE) |
| --- | --- | --- | --- | --- | --- | --- | --- |
|  | Risk of Bias | Inconsistency | Indirectness | Imprecision | Other |  |  |
| Body composition ^a^ | Serious ^k^ | Serious ^l^ | Not serious | Serious ^m^ | None | 51 (2 studies) | ⨁⨁◯◯LOW |
| Strength ^b^ | Serious ^k^ | Serious ^l^ | Not serious | Serious ^m^ | None | 118 (5 studies) | ⨁◯◯◯ VERY LOW |
| Speed ^c^ | Serious ^k^ | Serious ^l^ | Not serious | Serious ^m^ | None | 87 (4 studies) | ⨁◯◯◯ VERY LOW |
| Agility ^d^ | Serious ^k^ | Serious ^l^ | Not serious | Serious ^m^ | None | 87 (4 studies) | ⨁◯◯◯ VERY LOW |
| Power ^e^ | Serious ^k^ | Serious ^l^ | Not serious | Serious ^m^ | None | 128 (5 studies) | ⨁◯◯◯ VERY LOW |
| Endurance ^f^ | Serious ^k^ | Not serious | Not serious | Serious ^m^ | None | 27 (1 studies) | ⨁⨁◯◯LOW |
| Balance ^g^ | Serious ^k^ | Not serious | Not serious | Serious ^m^ | None | 30 (1 studies) | ⨁⨁◯◯LOW |
| Flexibility ^h^ | Serious ^k^ | Not serious | Not serious | Serious ^m^ | None | 20 (1 studies) | ⨁⨁◯◯LOW |
| Serve velocity ^i^ | Serious ^k^ | Serious ^l^ | Not serious | Serious ^m^ | None | 115 (4 studies) | ⨁◯◯◯ VERY LOW |
| Serve accuracy ^j^ | Serious ^k^ | Serious ^l^ | Not serious | Serious ^m^ | None | 64 (2 studies) | ⨁◯◯◯ VERY LOW |

a Outcome including Kraemer et al., 2000 and Kraemer et al., 2003; b Outcome including Kraemer et al., 2000; Kraemer et al., 2003; Zırhlı and Demirci, 2020; Wang et al., 2022; Ebada, 2022. c Outcome including Kraemer et al., 2003; Zırhlı and Demirci, 2020; Gül and Çelik, 2021; Cano´s et al., 2022. d Outcome including Bashir et al., 2019; Zırhlı and Demirci, 2020; Gül and Çelik, 2021; Cano´s et al., 2022. e Outcome including Kraemer et al., 2003; Zırhlı and Demirci, 2020; Gül and Çelik, 2021; Cano´s et al., 2022; Ebada, 2022. f Outcome including Kraemer et al., 2003. g Outcome including Bashir et al., 2019. h Outcome including Zırhlı et al., 2020. i Outcome including Kraemer et al., 2000; Kraemer et al., 2003; Cano´s et al., 2022; Wang et al., 2022. j Outcome including Fan, 2018; Wang et al., 2022. k Including study showed the some concerns or high risk of bias. Therefore, the certainty of the evidence was downgraded. l Including study showed inconsistent results with other studies. Therefore, the certainty of the evidence was downgraded. m Including study did not use power analysis to determine sample size. Therefore, the certainty of the evidence was downgraded.

GRADE Working Group grades of evidence High certainty: we are very confident that the true effect lies close to that of the estimate of the effect. Moderate certainty: we are moderately confident in the effect estimate: the true effect is likely to be close to the estimate of the effect, but there is a possibility that it is substantially different. Low certainty: our confidence in the effect estimate is limited: the true effect may be substantially different from the estimate of the effect. Very Low certainty: we have very little confidence in the effect estimate, the true effect is likely to be substantially different from the estimate of effect.
