# Supplementary material for: Effects of physical training programs on female tennis players’ performance: a systematic review and meta-analysis
Source: Front Physiol. 2023 Aug 17;14:1234114. doi: 10.3389/fphys.2023.1234114 (PMC10470022; doi:10.3389/fphys.2023.1234114)
Supplement: Supplementary file 2 [file Table2.DOCX]

| **Appendix 2** | **The data used for meta-analyses** | | | |  | | | | | |  | | |
| --- | --- | --- | --- | --- | --- | --- | --- | --- | --- | --- | --- | --- | --- |
| **Reference** | **Test** | **Experimental (pre-test)** | | | **Experimental (post-test)** | | | **Control (pre-test)** | | | **Control (post-test)** | | |
|  |  | **Mean** | **SD** | **n** | **Mean** | **SD** | **n** | **Mean** | **SD** | **n** | **Mean** | **SD** | **n** |
| Kraemer et al., 2000 EG1 | Strength (shoulder press,kg) | 27.17 | 5.85 | 8 | 36.18 | 7.85 | 8 | 26.35 | 4.68 | 4 | 26.23 | 3.98 | 4 |
|  | Serve velocity (km/h) | 27.63 | 3.97 | 8 | 35.49 | 4.54 | 8 | 25.20 | 2.92 | 4 | 24.64 | 3.57 | 4 |
| Kraemer et al., 2000 EG2 | Strength (shoulder press,kg) | 25.29 | 5.74 | 8 | 29.16 | 7.61 | 8 | 26.35 | 4.68 | 4 | 26.23 | 3.98 | 4 |
|  | Serve velocity (km/h) | 26.90 | 2.92 | 8 | 27.31 | 4.78 | 8 | 25.20 | 2.92 | 4 | 24.64 | 3.57 | 4 |
| Kraemer et al., 2003 EG1 | Strength (handgrip; N) | 335.7 | 40.8 | 9 | 361.9 | 34.5 | 9 | 330.6 | 40.3 | 4 | 316.9 | 46.1 | 4 |
|  | Speed (10m sprint; s) | 2.16 | 0.10 | 9 | 2.12 | 0.74 | 9 | 2.22 | 0.10 | 4 | 2.17 | 0.10 | 4 |
|  | Agility (LAT; s) | 7.07 | 0.95 | 9 | 7.43 | 0.22 | 9 | 7.70 | 0.64 | 4 | 7.60 | 0.48 | 4 |
|  | Power (CMJ,cm) | 40.27 | 6.53 | 9 | 63.01 | 9.12 | 9 | 40.37 | 6.08 | 4 | 43.24 | 6.25 | 4 |
|  | Serve velocity (km/h) | 27.50 | 4.04 | 9 | 35.26 | 5.47 | 9 | 24.56 | 4.68 | 4 | 22.58 | 3.33 | 4 |
| Kraemer et al., 2003 EG2 | Strength (handgrip; kg) | 321.3 | 46.8 | 10 | 356.1 | 44.5 | 10 | 330.6 | 40.3 | 4 | 316.9 | 46.1 | 4 |
|  | Speed (10m sprint; s) | 2.15 | 0.09 | 10 | 2.13 | 0.10 | 10 | 2.22 | 0.10 | 4 | 2.17 | 0.10 | 4 |
|  | Agility (LAT; s) | 7.33 | 1.14 | 10 | 7.49 | 0.42 | 10 | 7.70 | 0.64 | 4 | 7.60 | 0.48 | 4 |
|  | Power (CMJ,cm) | 40.20 | 6.93 | 10 | 55.91 | 7.43 | 10 | 40.37 | 6.08 | 4 | 43.24 | 6.25 | 4 |
|  | Serve velocity (km/h) | 26.78 | 3.80 | 10 | 30.59 | 3.88 | 10 | 24.56 | 4.68 | 4 | 22.58 | 3.33 | 4 |
| Fan, 2018 EG1 | Serve accuracy (points) | 39.4 | 9.8 | 8 | 53.3 | 10.9 | 8 | 53.4 | 11.6 | 4 | 50.7 | 6.7 | 4 |
| Fan, 2018 EG2 | Serve accuracy (points) | 34.8 | 10.3 | 8 | 52.1 | 12.1 | 8 | 53.4 | 11.6 | 4 | 50.7 | 6.7 | 4 |
| Zırhlı and Demirci, 2020 | Speed (10-m; s) | 2.25 | 0.21 | 10 | 2.10 | 0.20 | 10 | 2.46 | 0.24 | 10 | 2.49 | 0.23 | 10 |
|  | Power (VJH; cm) | 22.70 | 6.14 | 10 | 25.90 | 6.17 | 10 | 18.50 | 7.99 | 10 | 18.70 | 8.01 | 10 |
|  | Strength (hand grip; kg) | 23.85 | 3.76 | 10 | 27.45 | 4.38 | 10 | 20.94 | 4.18 | 10 | 21.01 | 4.62 | 10 |
|  | Agility (t test,s) | 13.44 | 0.433 | 10 | 12.35 | 0.479 | 10 | 13.92 | 0.847 | 10 | 13.86 | 0.898 | 10 |
| Gül and Çelik 2021 | Power (VJH; cm) | 26.68 | 1.71 | 8 | 27.93 | 1.81 | 8 | 26.25 | 1.84 | 8 | 26.56 | 1.57 | 8 |
|  | Speed (20 m sprint; s) | 4.07 | 0.07 | 8 | 3.88 | 0.06 | 8 | 4.07 | 0.07 | 8 | 4.06 | 0.07 | 8 |
|  | Agility (t test; s) | 12.37 | 3.75 | 8 | 11.22 | 3.45 | 8 | 11.98 | 2.99 | 8 | 11.93 | 0.26 | 8 |
| Cano´s et al., 2022 EG1 | Power (CMJ; cm) | 25.01 | 1.53 | 7 | 27.91 | 1.94 | 7 | 26.92 | 2.09 | 5 | 27.21 | 2.22 | 5 |
|  | Speed (10m; s) | 2.09 | 0.05 | 7 | 2.04 | 0.05 | 7 | 2.04 | 0.09 | 5 | 2.00 | 0.10 | 5 |
|  | Agility (5-0-5 test; s) | 2.89 | 0.10 | 7 | 2.84 | 0.09 | 7 | 2.99 | 0.09 | 5 | 2.96 | 0.05 | 5 |
|  | Serve velocity (km/h) | 125.14 | 5.24 | 7 | 129.43 | 6.78 | 7 | 113.33 | 13.67 | 5 | 117.67 | 11.55 | 5 |
| Cano´s et al., 2022 EG2 | Power (CMJ; cm) | 26.46 | 2.86 | 8 | 29.27 | 2.82 | 8 | 26.92 | 2.09 | 5 | 26.92 | 2.09 | 5 |
|  | Speed (10m; s) | 1.97 | 0.09 | 8 | 1.96 | 0.08 | 8 | 2.04 | 0.09 | 5 | 2.00 | 0.10 | 5 |
|  | Agility (5-0-5 test; s) | 2.83 | 1.10 | 8 | 2.78 | 0.07 | 8 | 2.99 | 0.09 | 5 | 2.96 | 0.05 | 5 |
|  | Serve velocity (km/h) | 138.5 | 8.05 | 8 | 141.13 | 12.21 | 8 | 113.33 | 13.67 | 5 | 117.67 | 11.55 | 5 |
| Wang et al., 2022 | Strength (abdominal fatigue test; s) | 155.8 | 42.04 | 12 | 163.05 | 46.11 | 12 | 145.1 | 56.45 | 11 | 149.34 | 35.78 | 11 |
|  | Serve velocity (km/h) | 120 | 21 | 12 | 131 | 11 | 12 | 121 | 21 | 11 | 125 | 15 | 11 |
|  | Serve accuracy (points) | 0.47 | 0.09 | 12 | 0.60 | 0.14 | 12 | 0.49 | 0.12 | 11 | 0.53 | 0.13 | 11 |

Note: VJH, vertical jump height; CMJ, countermovement jump; SLJ. Standing long jump; LAT, lateral agility test; EG, experimental group; CG, control group
